# Differentiating behavioral impact with or without vaccination certification under mass vaccination and non-pharmaceutical interventions on mitigating COVID-19

**DOI:** 10.1038/s41598-023-50421-9

**Published:** 2024-01-06

**Authors:** Hu Cao, Longbing Cao

**Affiliations:** School of Computing, Macquarie University, Sydney, NSW 2109 Australia

**Keywords:** Risk factors, Mathematics and computing

## Abstract

As COVID-19 vaccines became widely available worldwide, many countries implemented vaccination certification, also known as a “green pass”, to promote and expedite vaccination on containing virus spread from the latter half of 2021. This policy allowed those vaccinated to have more freedom in public activities compared to more constraints on the unvaccinated in addition to existing non-pharmaceutical interventions (NPIs). Accordingly, the vaccination certification also induced heterogeneous behaviors of unvaccinated and vaccinated groups. This makes it essential yet challenging to model the behavioral impact of vaccination certification on the two groups and the transmission dynamics of COVID-19 within and between the groups. Very limited quantitative work is available for addressing these purposes. Here we propose an extended epidemiological model SEIQRD$^2$ to effectively distinguish the behavioral impact of vaccination certification on unvaccinated and vaccinated groups through incorporating two contrastive transmission chains. SEIQRD$^2$ also quantifies the impact of the green pass policy. With the resurgence of COVID-19 in Greece, Austria, and Israel in 2021, our simulation results indicate that their implementation of vaccination certification brought about more than a 14-fold decrease in the total number of infections and deaths as compared to a scenario with no such a policy. Additionally, a green pass policy may offer a reasonable practical solution to strike the balance between public health and individual’s freedom during the pandemic.

## Introduction

In the early stage of COVID-19, non-pharmaceutical interventions (NPIs) were the only effective way to contain its spread^1,2^. Stringent preventative measures, such as limitation on social gatherings, shutdown of international borders, travel prohibition, night-time curfew, and lockdown, were implemented to thwart the spread of COVID-19^1^. With the effective vaccines available against COVID-19, many countries initiated their nationwide vaccination campaigns^3^, including the UK, the US, Israel, and European countries. Until the second half of 2021, the vaccines were widely available around the world. Subsequently, a vaccine passport or certificate scheme was implemented in numerous nations, which enabled people and companies with proof of vaccination to enjoy unrestricted access to certain amenities and activities. The vaccination certification encouraged people to receive vaccination and accelerated mass vaccination. For example, Israel was one of the first countries to launch the nationwide vaccination campaign and carry out the green pass policy^4^.

To analyze the spread of COVID-19 and forecast its potential development, researchers have made significant efforts to model the virus behaviors and impact^5^. A typical modeling method is epidemiological models, which can be seen as a reliable, efficient and persuasive solution for airborne pandemics, including COVID-19^6^. As a classic epidemic model, susceptible-infected-removed (SEIR) and its extended versions provide a clear explanation of COVID-19 and forecast its spread. To understand the impact of COVID-19, researchers initially focused on studying the effectiveness of different NPIs on the transmission of the virus^7–11^. For example, Jonas et al.^7^ discussed the effectiveness of interventions under varying implementation and timing. They showed that strict NPIs with early implementation could largely reduce the spread of COVID-19. Seth et al.^8^ conducted an assessment of a range of NPIs in Europe. Their results demonstrated that lockdowns have a huge impact on mitigating COVID-19 transmission. As effective vaccines for COVID-19 were available, increasing concern was made on strategies for distributing the vaccines and balancing an optimal combination of vaccination and NPIs. As a result, a substantial number of techniques and approaches were suggested to investigate optimal distribution plans and combinations of vaccination and NPIs^2,12–23^. Yang et al.^12^ argued that equitable access to vaccines would have a lasting beneficial effect on controlling COVID-19 globally. Sam et al.^13^ examined different mixtures of vaccinations and NPIs, while changing the effectiveness of COVID-19 vaccines. They concluded that vaccination alone is insufficient to contain the COVID-19 epidemic resurgence. Further, Matrajt et al.^20^ investigated various vaccination strategies aimed at lowering the overall number of cases. Ge et al.^17,24^ evaluated the effectiveness of all kinds of NPIs and vaccination in controlling COVID-19, respectively. Sonabend et al.^22^ assessed the roadmap of England by considering the lifting of NPIs and vaccination roll-out under the influence of the COVID-19 pandemic. Dan et al.^23^ proposed some concrete recommendations to ensure equity in the distribution of vaccines.

The implementation of the green pass policy differentiated people’s behaviors within and between vaccinated and unvaccinated groups. Therefore, from a behavior informatics perspective, it is important to measure such behavioral difference and impact caused by vaccination certification on the dynamics of COVID-19^5,25^. However, how to characterize heterogeneous behaviors of vaccinated versus unvaccinated groups into epidemiological models is still an open issue. Limited work is available in evaluating the behavior difference between vaccinated and unvaccinated groups in evaluation of the effectiveness of the green pass policy on COVID-19 transmission from a quantitative perspective. In late July 2022, the Oxford COVID-19 Government Response Tracker (OxCGRT) distinguished between the control measures for vaccinated individuals and those for unvaccinated individuals (https://www.bsg.ox.ac.uk/research/covid-19-government-response-tracker) due to the introduction of the green pass policy. This provides some data for the evaluation.

Despite some available studies^26–28^ exploring the effects of diverse behaviors on COVID-19 dynamics, these were not utilized in countries with the green pass policy in place. A vaccine green pass (vaccine passport or certification) is a digital or paper record demonstrating that its holders have been absolutely vaccinated or have recovered after having contracted COVID-19. It grants its holders more freedom of gathering and movement than unvaccinated individuals. With the reemergence of the pandemic, people who are not vaccinated are limited in their social activities, but those with green passes are allowed to access various public establishments, such as restaurants, bars, cafés, and indoor venues^29^. Cliff et al.^26^ modeled the development of COVID-19 by incorporating different human behavior patterns under varying environments, such as schools, homes, and workplaces. A study conducted by Teddy et al.^28^ focused on population diversity in terms of age, vulnerability, and complications of COVID-19. However, these methods are unable to distinguish the vaccination status of the population and have not taken into account the effects of the green pass policy on COVID-19 transmission.

Given that the presence of vaccination certification fosters different behaviors between unvaccinated and vaccinated groups, COVID-19 modeling^5^ may benefit from involving behavioral heterogeneity and their impact on COVID-19 transmission from a behavior informatics perspective^25^. With this motivation, we propose a modified SEIR epidemiological model SEIQRD$^2$. In contrast to the past research that simply examined the risk of vaccination certificate policy on vaccinated and unvaccinated individuals^30,31^, SEIQRD$^2$ goes further by measuring the human behavioral disparity between unvaccinated and vaccinated groups utilizing two dependent SEIR-based transmission chains. The two transmission chains refer to the spread of COVID-19 among the unvaccinated and vaccinated individuals, respectively. Besides, SEIQRD$^2$ provides a novel mechanism to describe the actual interaction between the two groups by the interplay between the two transmission chains. Based on the innovative design of SEIQRD$^2$, it could be used to evaluate the efficiency of green pass policy on COVID-19 from the epidemiological perspective, which addresses the gap in exploring the effectiveness of green pass policy using quantitative methods.

In addition, our model recognizes that the effectiveness of vaccination decreases and that immunity weakens during the COVID-19 pandemic^32,33^. Differently, many research studies on COVID-19 do not consider the decreasing effect of vaccination on infection^17,18,34^. This ignorance may conflict with the reality of the COVID-19 resurgence, to which the waning effectiveness of vaccines made a contribution^35–38^.

This work explores the above open questions per the following real-world scenarios and assumptions. First, authorities would relax NPIs and grant the vaccinated group more freedom to activities such as travel and social gatherings than the unvaccinated^29^. Second, the COVID-19 resurgence could be triggered after the implementation of the green pass policy. These assumptions represent the practical situations in the countries that implemented the green pass policy in 2021.

SEIQRD$^2$ analyzes the mentioned features and addresses the limitations in modeling viral and human behavior patterns during mass immunization, as well as the effects of vaccination documentation and NPIs. SEIQRD$^2$ is able to illustrate the contrast between behaviors of those who are vaccinated and those who are not under the green pass policy as a result of our design of two related transmission chains. At last, we show the accuracy of the SEIQRD$^2$ model in representing COVID-19 transmission in three pioneering countries with vaccination documentation: Greece, Austria, and Israel.

## Results

### Datasets

The data used in this study includes epidemiological data, vaccination information, NPIs events, and data about virus variants. These datasets are publicly available. For instance, the epidemiological data are sourced from the Johns Hopkins University Center for Systems Science and Engineering (JHU CSSE)^39,40^, which includes daily statistics on confirmed cases and deaths. The vaccination information is downloaded from Our World in Data^41^. The Oxford COVID-19 Government Response Tracker (OxCGRT)^42^ provides the NPIs information and CoVariants^43^ with viral variants.

Besides, these datasets are selected from the latter part of 2021 for two distinct reasons. Firstly, an increasing number of countries started to implement green pass policies since the second half of 2021. In early 2022, due to the emergence of a new viral straint, Omicron, many countries retracted their green pass policies and loosened control measures even for those who were not vaccinated. Secondly, most countries also relaxed COVID-19 monitoring or stopped to record daily cases in late December 2021, which led to the inconsistency of confirmed cases. Based on these reasons, the data from July to December 2021 are suitable for our research purpose. The data and code can be found on GitHub (https://github.com/lzxiaohu/SEIQRD2).

We apply SEIQRD$^2$ in three countries: Greece, Austria, and Israel. The three countries are selected for the following reasons. First, these countries experienced a whole wave of COVID-19 caused by the Delta variant when green pass policy came into effect. For example, the resurgence of COVID-19 in Greece and Austria lasted from mid-September 2021 to mid-December 2021. Israel suffered from a resurgence of COVID-19 from late July 2021 to late October 2021. It is helpful for us to test the validity of the model SEIQRD$^2$. Second, the three countries are distributed on different continents and hold different ethnic and cultural backgrounds, which promote their varied behaviors and responses to NPIs, vaccination, and green pass policy. Their data are thus representative to evaluate the performance of our model across regions. Besides, Greece, Austria, and Israel are those of first countries to carry out green pass policy. There is more detailed information about their records of green pass policies.

### Greece

**Figure 1:**
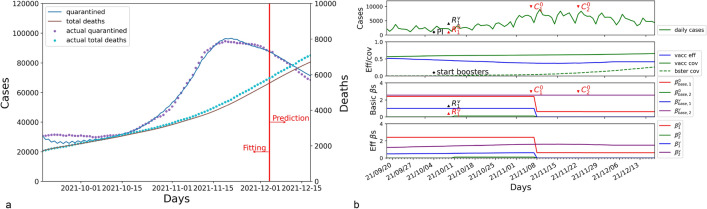
The results of SEIQRD$^2$ in Greece for the period between 18 September 2021 and 18 December 2021. (**a**) refers to the modeling results for the quarantined and total deaths in Greece, where the blue line refers to the quarantined cases estimated by SEIQRD$^2$, and the brown line refers to the total death cases produced by our method. (**b**) displays the impact of external factors during the COVID-19 resurgence. The first subplot from the top refers to the daily reported cases in Greece. Vaccination coverage, booster coverage, and the efficacy of vaccination are shown in the second subplot. The third subplot indicates the impact of NPIs on the transmission of the COVID-19 wave, in which the red line refers to the basic transmission rate $\beta _{base,1}^{0}$ in the unvaccinated group, the green line refers to the basic transmission rate $\beta _{base,2}^{0}$ among the unvaccinated caused by the vaccinated, the blue line represents the basic transmission rate $\beta _{base,1}^{v}$ among the vaccinated group caused by the unvaccinated people, and the purple line shows the basic transmission rate $\beta _{base,2}^{v}$ among the vaccinated. The fourth subplot shows the joint impact of both NPIs and vaccination during the COVID-19 resurgence, which are denoted by the four effective transmission rates $\beta _{1}^{0}$, $\beta _{2}^{0}$, $\beta _{1}^{v}$, and $\beta _{2}^{v}$. PI represents the start of the booster campaign. $\{ R_1^{0}, C_1^{0}, C_2^{0}\}$ denote the NPI events for the unvaccinated, where *R* means the relaxation of an NPI and *C* refers to the control of an NPI. Similarly, $\{R_1^{v}\}$ is the NPI event for the vaccinated. Their physical meanings are also listed in the appendix.

From September 2021 to December 2021, Greece experienced a new resurgence caused by the Delta variant. Figure 1a displays the forecasting results about quarantined cases and total deaths for the next 14 days from 05 December to 18 December 2021. The prediction accuracy is measured by the metric MAPE, achieving 3.229% for quarantined cases and 4.853% for death cases. Figure 1b shows the combined impact of NPIs and mass vaccination during the COVID-19 resurgence.

As shown in Fig. 1a, the turning point of the COVID-19 wave appeared on 09 November 2021, which coincided with the implementation of the NPI event $C_1^{0}$ that required unvaccinated individuals to show a negative COVID-19 test result before entering all indoor public places. The NPI event $C_1^{0}$ obviously reduced the effective transmission rates $\beta _{1}^{0}$ and $\beta _{1}^{v}$ from 2.418 and 0.482 to 0.625 and almost 0 by restricting the physical contact between the two groups. The reduction of $\beta _{1}^{0}$ and $\beta _{1}^{v}$ responds to the decrease of the basic transmission rates $\beta _{base, 1}^{0}$ and $\beta _{base, 1}^{v}$, which further demonstrates the effectiveness of $C_1^{0}$ for the unvaccinated group. The following NPI event $C_{2}^{0}$ that banned unvaccinated individuals from accessing restaurants came into effect on 22 November 2021. $C_{2}^{0}$ further restricted the social activities of those unvaccinated, leading to the decline of transmission rate $\beta _{1}^{0}$. Combined $\{ \beta _{1}^{v},\beta _{2}^{v} \}$ with $\{ \beta _{base, 1}^{v},\beta _{base, 2}^{v} \}$, these NPI events did not obviously change the dynamics of the COVID-19 among the green pass holders. In order to explain the impact of NPIs in detail, Table 1 lists their contribution to the four couples of transmission rates during the COVID-19 resurgence. Table 1 illustrates the effectiveness of the green pass policy by restricting the unvaccinated group.

**Table 1:** Attributes of NPI impact in Greece.

NPI events	Basic transmission rates				Effective transmission rates			
	$\beta _{base, 1}^{0}$	$\beta _{base, 2}^{0}$	$\beta _{base, 1}^{v}$	$\beta _{base, 2}^{v}$	$\beta _{1}^{0}$	$\beta _{2}^{0}$	$\beta _{1}^{v}$	$\beta _{2}^{v}$
Initial values	2.418	0.001	1.000	2.577	2.418	0.001	0.482	1.164
$R_{1}^{0}$: 09 Oct. 2021 $R_{1}^{v}$:	2.418	0.102	1.000	2.577	2.418	0.102	0.541	1.394
$C_{1}^{0}$: 06 Nov. 2021 –:	0.625	0.001	0.001	2.577	0.625	0.001	0.001	1.592
$C_{2}^{0}$: 22 Nov. 2021 –:	0.531	0.001	0.001	2.577	0.531	0.001	0.001	1.599

On the other hand, the green pass policy accelerated the mass booster vaccination shown in Fig. 1b. The booster campaign enhanced the efficacy of vaccination from 0.373 (15 November 2021) to about 0.500 (06 December 2021), contributing to the mitigation of COVID-19 among the vaccinated individuals.

According to the results of SEIQRD$^2$, the mitigation of the COVID-19 wave may be attributed to both control measures and booster shots. In Greece, NPI events were used to reduce the COVID-19 transmission in the unvaccinated group, whereas vaccine booster was helpful for the vaccinated individuals.

### Austria

**Figure 2:**
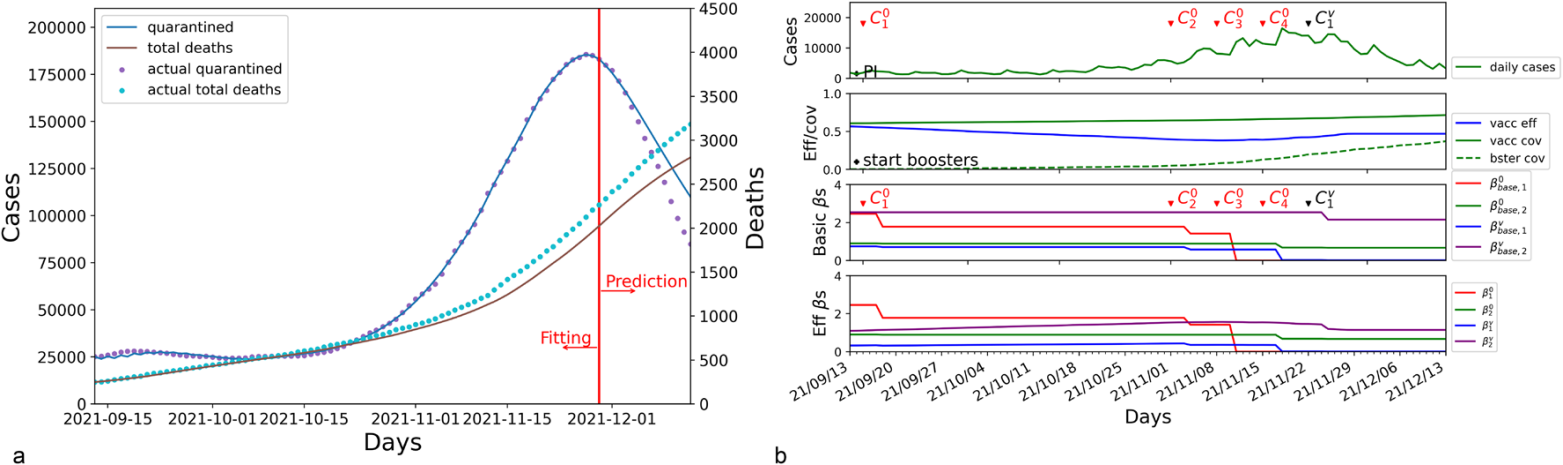
The results of SEIQRD$^2$ in Austria for the period between 13 September 2021 and 13 December 2021. (**a**) is the modeling results for the quarantined and total deaths in Austria. (**b**) displays the impact of external factors during the COVID-19 resurgence.

The modeling results for Austria are shown in Fig. 2. A resurgence with more infected cases occurred in the middle of September 2021. SEIQRD$^2$ predicts the dynamics of the COVID-19 wave for the next 14 days, showing 5.411% MAPE in forecasting quarantined cases, as shown in Fig. 2a. Figure 2b displays the joint impact of external factors.

The wave dynamics could be clearly explained by SEIQRD$^2$ as shown in Fig. 2b. After the peak daily cases reported on 18 November 2021, the wave reached a plateau and then declined sharply on 29 November 2021. It was closely associated with two lockdowns taking place on 15 November 2021 and 22 November 2021. In the first lockdown, the government of Austria required all unvaccinated individuals to stay at home ($C_{4}^{0}$), which reduced the physical contact between the two groups. It lowered the effective transmission rate $\beta _{2}^{0}$ from 0.999 to 0.632. After that, their authorities carried out the second lockdown ($C_{1}^{v}$) to restrict the social activities of those green pass holders. This second lockdown reduced the values of $\beta _{2}^{0}$ and $\beta _{2}^{v}$ from 0.632 and 1.588 to 0.345 and 1.048, corresponding to 0.345 of $\beta _{base, 2}^{0}$ and 1.817 of $\beta _{base, 2}^{v}$. Compared to the first lockdown enforced on the unvaccinated group, the second one on green pass holders made a greater contribution to flattening the wave. It implied that the resurgence may be caused by the over-relaxation of NPIs, especially for those green pass holders. The impact of other NPI events is listed in Table 2.

**Table 2:** Attributes of NPI impact in Austria.

NPI events	Basic transmission rates				Effective transmission rates			
	$\beta _{base, 1}^{0}$	$\beta _{base, 2}^{0}$	$\beta _{base, 1}^{v}$	$\beta _{base, 2}^{v}$	$\beta _{1}^{0}$	$\beta _{2}^{0}$	$\beta _{1}^{v}$	$\beta _{2}^{v}$
Initial values	2.365	1.000	0.487	2.607	2.365	1.000	0.211	1.130
$C_{1}^{0}$: 15 Sep. 2021 –:	1.915	0.999	0.487	2.607	1.915	0.999	0.214	1.145
$C_{2}^{0}$: 01 Nov. 2021 –:	1.915	0.999	0.070	2.607	1.915	0.999	0.042	1.577
$C_{3}^{0}$: 08 Nov. 2021 –:	1.915	0.999	0.001	2.607	1.915	0.999	0.001	1.610
$C_{4}^{0}$: 15 Nov. 2021 –:	1.915	0.632	0.001	2.607	1.915	0.632	0.001	1.588
–: $C_{1}^{v}$: 22 Nov. 2021	1.915	0.345	0.001	1.817	1.915	0.345	0.001	1.048

During the resurgence, vaccination also played a crucial role in the mitigation of COVID-19. As the resurgence worsened, booster shots received greater attention in public discourse, and booster coverage grew substantially from November 2021. Until 13 December 2021, it improved vaccination effectiveness from the lowest point of 0.382 to 0.551. After the second lockdown, the effective transmission rate $\beta _{2}^{v}$ gradually decreased from 1.048 to 0.816 $(1.817*(1-0.551))$ because of the increasing effectiveness of vaccination.

The results of SEIQRD$^2$ demonstrate that proper NPIs may still be essential for the vaccinated group, especially when the efficacy of vaccination decreases. At the same time, vaccination could be also critical to control the spread of COVID-19.

### Israel

**Figure 3:**
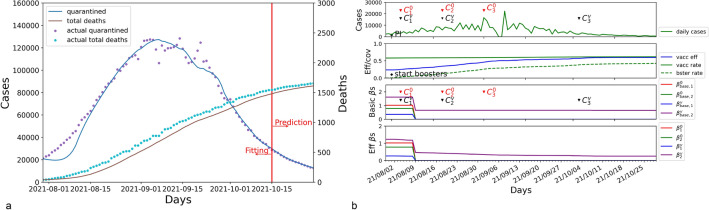
The results of SEIQRD$^2$ in Israel for the period between 30 July 2021 and 30 October 2021. (**a**) is the modeling results for the quarantined and total deaths in Israel. (**b**) is the impact of external factors on the COVID-19 resurgence.

Israel was attacked by another wave from August 2021 to October 2021. Figure 3 illustrates the modeling results for Israel. The results modeled by SEIQRD$^2$ match the dynamics of this viral resurgence. The MAPE for forecasting quarantined cases is 1.292%, and the MAPE for estimating the total deaths is 3.167%. Figure 3b shows the combined impact of NPIs and mass vaccination during the COVID-19 resurgence in Israel.

Figure 3b elucidates the course of the resurgence by the transmission rates of COVID-19. At the initial outbreak of the wave, the Israeli government took stringent NPI measures to control the spread of viral infections. These control measures include showing a negative COVID-19 test result for those unvaccinated ($C_{1}^{0}$) and limiting the gatherings of green pass holders ($C_{1}^{v}$), which substantially reduced the four basic transmission rates $\beta _{1}^{0}$, $\beta _{2}^{0}$, $\beta _{1}^{v}$, and $\beta _{2}^{v}$. For example, $\beta _{1}^{0}$ and $\beta _{2}^{v}$ decreased radically from 1.024 and 1.236 to 0.001 and 0.486, respectively. It means that only green pass holders can participate in social activities with limitations, whereas the unvaccinated people were required to stay home. That accords with the practical circumstances in Israel during the re-emergence of the virus. As a consequence, the physical contact between the two groups declined substantially, which is demonstrated by the reduction of $\beta _{base, 2}^{0}$ and $\beta _{base, 1}^{v}$ from 0.779 and 0.350 to 0.001 and 0.001. The impact of NPI events is shown in Table 3.

**Table 3:** Attributes of NPI impact in Israel.

NPI events	Basic transmission rates				Effective transmission rates			
	$\beta _{base, 1}^{0}$	$\beta _{base, 2}^{0}$	$\beta _{base, 1}^{v}$	$\beta _{base, 2}^{v}$	$\beta _{1}^{0}$	$\beta _{2}^{0}$	$\beta _{1}^{v}$	$\beta _{2}^{v}$
Initial values	1.024	0.779	0.350	1.621	1.024	0.779	0.112	1.236
$C_{1}^{0}$: 04 Aug. 2021 $C_{1}^{v}$:	0.001	0.001	0.001	0.648	0.001	0.001	0.001	0.486
$C_{2}^{0}$: 18 Aug. 2021 $C_{2}^{v}$:	0.001	0.001	0.001	0.647	0.001	0.001	0.001	0.425
$C_{3}^{0}$: 01 Sep. 2021 –:	0.001	0.001	0.001	0.647	0.001	0.001	0.001	0.348
–: $C_{3}^{v}$: 03 Oct. 2021	0.001	0.001	0.001	0.637	0.001	0.001	0.001	0.274

In the meantime, Israel started its mass booster campaign to strengthen the effectiveness of vaccination. The turning point of daily cases appeared when the efficacy of vaccination rose from 0.236 to 0.596. Until the end of COVID-19 resurgence, the value of $\beta _{2}^{v}$ was $0.257 (0.637*(1-0.596))$. Therefore, accelerating boosters in a short time played an important role in suppressing the spread of this COVID-19 wave. Subsequently, Israel effectively managed the COVID-19 resurgence with the amalgamation of effectual NPIs and widespread booster vaccination in a span of three months.

### Analyses

To quantify the effect of the green pass policy, we compare the total number of confirmed cases and deaths during the implementation of the policy with the number of infected and deceased individuals when unvaccinated people were granted the same freedom as vaccinated individuals. The detailed information can be found in Table 4. This simulated result indicates that the green pass policy would decrease the total confirmed cases and fatalities by more than 14 times in all three countries. Furthermore, it quantifies the effect of the green pass policy from a quantitative perspective and provides empirical evidence of enabling vaccination certification.

**Table 4:** The comparison of with and without green pass policy in Greece, Austria, and Israel.

Country	Total infected			Deceased		
	Green pass policy	Without	$\Delta _{1}$ (%)	Green pass policy	Without	$\Delta _{2}$ (%)
Greece	509,968	7,755,818	$+1420$	6738	123,547	$+1733$
Austria	388,373	6,283,800	$+1518$	2781	47,129	$+1595$
Israel	468,273	7,855,803	$+1577$	1612	26,101	$+1519$

Further, the green pass policy could be a reasonable and practical solution to achieve the balance between public health and individual’s freedom of behaving. According to the experimental results, the vaccinated group in the three countries always experienced greater freedom compared to the unvaccinated individuals. This is evidenced by the basic transmission rates $\beta _{base, 1}^{0}$ and $\beta _{base, 2}^{v}$. Nevertheless, the COVID-19 resurgence could be controlled successfully by mass booster and proper restriction for the unvaccinated. For instance, the unvaccinated group in Greece had restrictions on certain social activities in order to maintain a transmission rate of 0.625, while the vaccinated group had more relaxation corresponding transmission rate of 2.577. Similar situations happened in Israel and Austria. Compared to the restrictions for all people before the first second half of 2021, the green pass policy ensures the freedom of vaccinated individuals, who make up more than 60% of the total population. At the same time, it allows the country to restart its national economy and resume the physical educational activities.

On the other hand, all three countries, Greece, Austria, and Israel, took reasonable control measures to combat COVID-19. This set of actions involve mass vaccination and customized NPIs. As displayed in the results of the three countries, booster shots and NPIs indeed played a crucial role in preventing the transmission of COVID-19. For example, Israel was the most successful one among these countries in stopping the spread of COVID-19. In the early stage of resurgence, the Israeli government carried out a series of NPIs ($C_{1}^{0}-C_{3}^{0}$, $C_{1}^{v}-C_{3}^{v}$) for the unvaccinated and green pass holders, respectively. After that, a nationwide booster campaign was accelerated, and the booster coverage increased quickly from 0.5 to 24.5% within a month, improving vaccination efficacy from 0.236 to 0.45. Greece and Austria adopted a similar strategy to control the spread of COVID-19 (Figs. 1 and 2).

In summary, the green pass policy is a reasonable and scientific solution to cope with the COVID-19 pandemic by allowing the public freedom of the vaccinated groups. It enables a trade-off between public health and individual’s public freedom by reducing the total number of infections and deaths. It could be used as a strategy to avoid a potential pandemic and full lockdown.

### Ablation study

To further evaluate the performance of our method SEIQRD$^2$, we conducted three ablation studies in Greece, Austria, and Israel. The first ablation study involves creating a variant of the SEIQRD$^2$ model called SPEIQRD. This variant does not take into account the green pass policy and assumes that both groups have the same behavior patterns. By leaving out the interaction between the vaccinated and unvaccinated individuals in SEIQRD$^2$, the model, SVEIQRD, is produced. The third ablation involves deriving the model SEIQRD$^2_c$ from SEIQRD$^2$ by assuming that the vaccination efficacy remains constant.

Accordingly, we generate three variants of our SEIQRD$^2$: SPEIQRD, SVEIQRD, and SEIQRD$^2_c$ for the ablation study. The information about these different versions can be found in the Supplementary Information. In addition, the conclusion of the ablation study is based on independent parameters. The least-squares method is employed by the optimization algorithm to obtain the parameter values.

**Figure 4:**
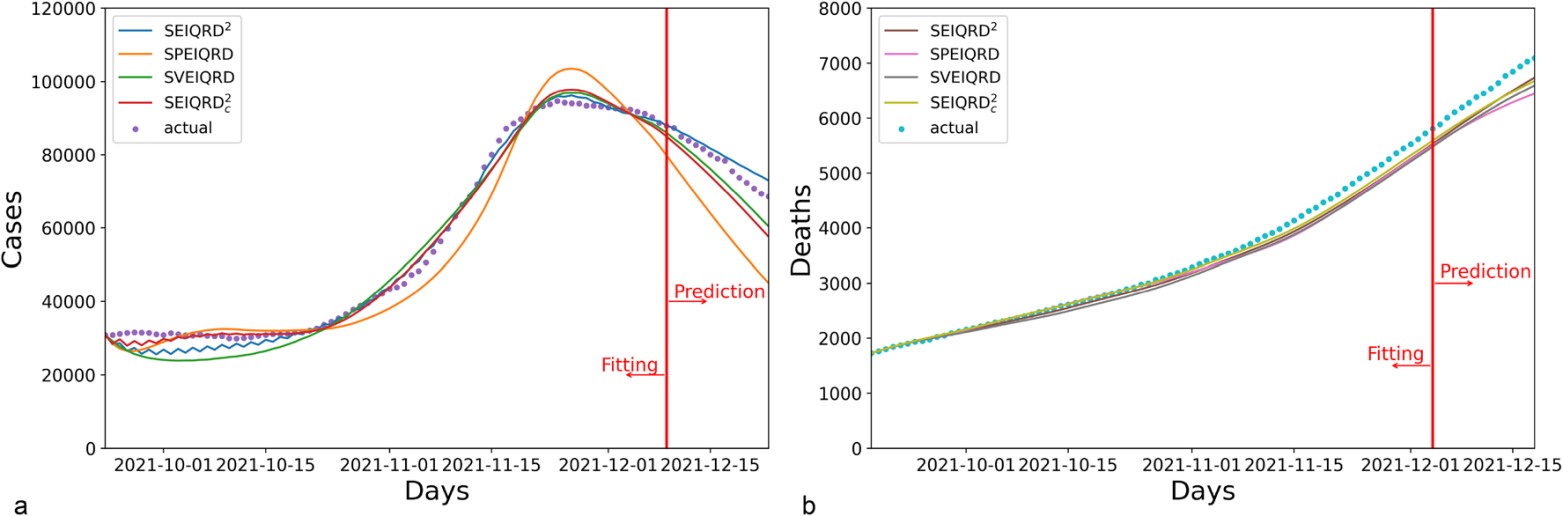
The results of four variants of our method SEIQRD$^2$ in Greece. In the four variants of method, SPEIQRD ignores the impact of enforcing vaccination certification. SVEIQRD overlooks the interactions between unvaccinated and vaccinated groups. SEIQRD$^2_c$ neglects the waning effectiveness of vaccine.

The simulation results for Greece are presented in Fig. 4. Compared to the three variants SPEIQRD, SVEIQRD, and SEIQRD$^2_c$, the full model SEIQRD$^2$ provides more accurate forecasting of the number of quarantined cases, and its results for the number of deaths closely align with the actual values.

**Figure 5:**
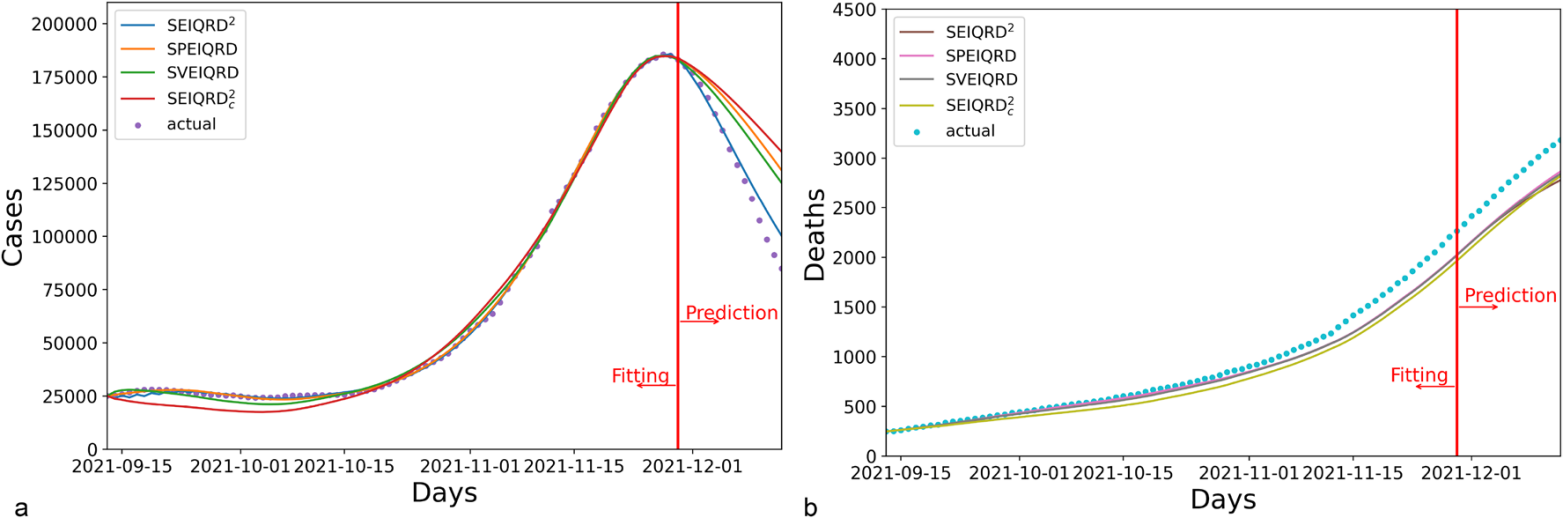
The results of four variants of our method SEIQRD$^2$ in Austria.

The results for Austria are shown in Fig. 5. The three variants SPEIQRD, SVEIQRD, and SEIQRD$^2_c$ achieve the MAPE of 20.896%, 17.445%, and 24.326% respectively, while SEIQRD$^2$ achieves an MAPE of 5.411% in forecasting the quarantined cases of the next 14 days. SEIQRD$^2$’s evident advantage over the three variants demonstrates the significant role of NPI, vaccination, and the interaction between unvaccinated and vaccinated groups in mitigating the spread of COVID-19.

**Figure 6:**
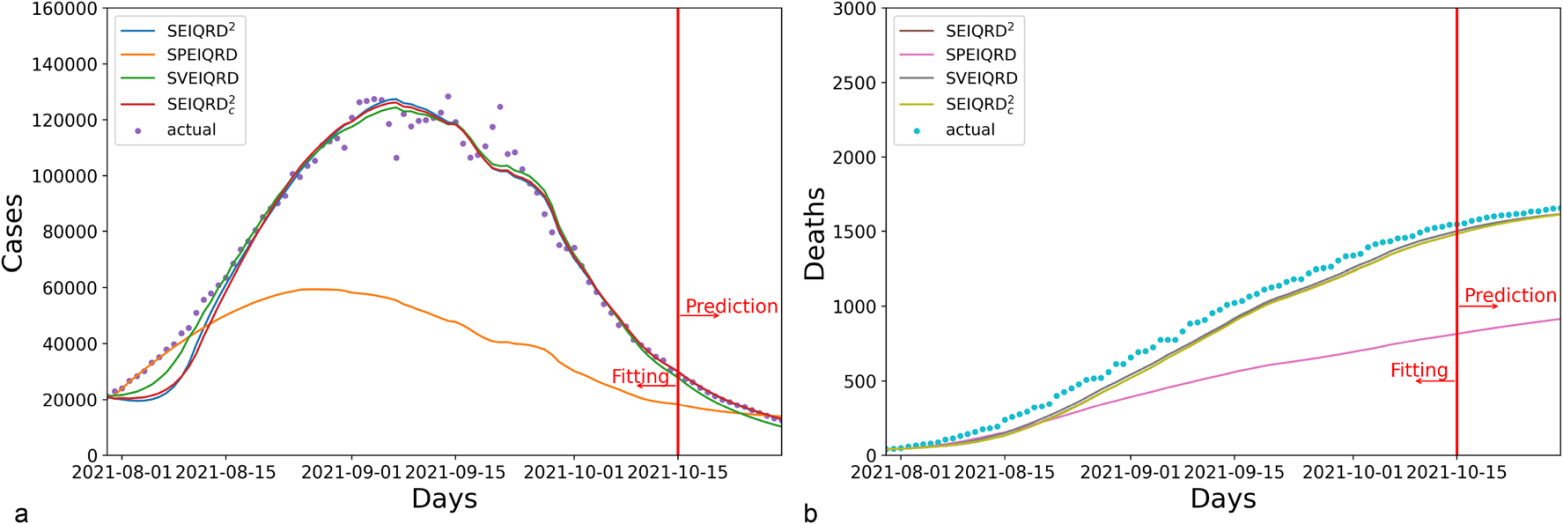
The results of four variants of our method SEIQRD$^2$ in Israel.

Figure 6 displays the results for Israel. The results of SPEIQRD significantly deviate from the realistic situation, further demonstrating that the green pass policy plays a leading role in containing the COVID-19 resurgence in Israel. Israel was the first country to carry out the green pass policy. Compared with Greece and Austria, Israel heavily relied on the green pass policy to contain the resurgence. That explains why the results of SPEIQRD are much worse. Among all four methods, SEIQRD$^2$ obtains the least error in predicting the quarantined cases, whose MAPE is 1.292%.

Table 5 shows the forecasting performance of all models in terms of MAPE. It demonstrates that SEIQRD$^2$ is more effective than its counterpart SVEIQRD, suggesting that interactions between different groups are crucial in modeling the progression of COVID-19. More importantly, it supports that the innovative design of two related transmission chains are reasonable and convincing by comparing SEIQRD$^2$ and SVEIQRD. By contrast, the underwhelming performance of SPEIQRD highlights the significance of the green pass policy in simulating COVID-19. Although the MAPE results of both SEIQRD$^2_c$ and SEIQRD$^2$ in predicting total deaths are similar, SEIQRD$^2$ makes more accurate prediction of quarantined cases than SVEIQRD. This endorses the fact that the reduced efficacy of the vaccine is a key element in modeling the COVID-19 pandemic.

**Table 5:** The MAPE performance of the next 14-day prediction of cases in Greece, Austria, and Israel for the ablation study.

Country	SEIQRD$^2$		SPEIQRD		SVEIQRD		SEIQRD$^2_c$	
	Quarantined (%)	Deceased (%)	Quarantined (%)	Deceased (%)	Quarantined (%)	Deceased (%)	Quarantined (%)	Deceased (%)
Greece	**3.229**	4.853	22.626	7.014	6.289	6.184	8.989	**4.676**
Austria	**5.411**	11.133	20.896	**9.999**	17.445	10.492	24.326	12.107
Israel	**1.292**	3.167	18.347	45.994	13.157	**2.557**	1.825	3.208

Significant values are in bold.

In conclusion, these ablation studies convincingly illustrate the importance of involving the green pass policy by considering the difference between the behaviors of unvaccinated and vaccinated groups and their interactions. Beyond the heterogeneous behaviors, our method emphasizes the contribution of the waning efficacy of vaccination to modeling the COVID-19 infection.

## Discussion

In this work, we develop a deterministic compartmental model SEIQRD$^2$ to characterize the effect of the green pass policy on the dynamics of COVID-19, in particular, reshaping people’s public behaviors of vaccinated vs unvaccinated groups. In comparison to the prior SEIR-based models for COVID-19, SEIQRD$^2$ accounts for the distinction of behaviors between vaccinated and unvaccinated groups in public activities and how these different behaviors interrelate by introducing a novel design of two interlinked transmission chains corresponding to the behaviors of vaccinated and unvaccinated groups. Further, SEIQRD$^2$ recognizes the waning effect of vaccination by introducing the decay curve of vaccination immunity. We test SEIQRD$^2$ in examining the revivals of three countries due to the coronavirus Delta strain and predicting the progress of COVID-19 in the next 14 days. Our findings demonstrate that SEIQRD$^2$ quantifies the resurgence of COVID-19 in Greece, Austria, and Israel during the latter part of 2021 better than other SEIR models without considering the effect of vaccination certification and its reshaping effect on the behaviors of vaccinated and unvaccinated groups. The experimental results suggest that SEIQRD$^2$ characterizes the propagation of COVID-19 under the green pass policy, taking into account the difference of behaviors between unvaccinated and vaccinated individuals, their interactions within their groups, and the decreasing effectiveness of vaccination. Furthermore, the impact of these three factors is assessed through ablation studies, confirming the credibility of our approach.

The study in this work suggests that the green pass policy indeed played a crucial role in mitigating COVID-19 by introducing vaccination certification. The green pass policy could not only reduce the total number of infections and deaths more than 14-fold in comparison with those unvaccinated but also achieve a reasonable balance between public health and individual’s freedom in public. Vaccination certification provides evidence-based guide for health authorities to restart their national economy and resume educational activities to normal. During the resurgence of COVID-19, mass booster and proper restriction for unvaccinated group could be useful measures to control its spread between vaccinated and unvaccinated. In cases of extreme contagion, such as a super-spreader, it would still be essential for control measures to be implemented among the vaccinated group.

This study is subject to several limitations. Firstly, the model parameters, such as effective transmission rates, are described by specific values rather than probability distributions. In the following work, we plan to quantify the transmission rates by incorporating specific statistical methods into SEIQRD$^2$. Secondly, the existing model ignores the number of hospitalized people, which may be another critical factor in deciding when to upgrade control measures. Once these data are available, further research could be made. Thirdly, the model assumes all infected individuals are detected and confirmed, which may be an ideal case. In reality, some infected people may be asymptomatic and recover without reporting or medical confirmation. This proportion could be small in the three developed countries, so it may not challenge the general results of the method. At last, the need for more information about the vaccination status of confirmed cases makes it hard to precisely reflect the effective transmission rates among the vaccinated group. This problem may be leveraged once the vaccination status information is available. Even though, the values of the effective transmission rates disclose some insights into the role of the green pass policy in the COVID-19 pandemic.

Overall, this work is one of the first attempts to address the impact of the green pass policy on the COVID-19 transmission from a behavior informatics perspective. It incorporates the behavior heterogeneity of unvaccinated and vaccinated groups into the SEIR-based modeling. This work could be further extended to generate “what-if” scenario analysis and provide decision-makers with quantitative results on how appropriate NPIs and vaccination policies could look like in containing future waves of infection.

## Methods

### Model structure and assumptions

**Figure 7:**
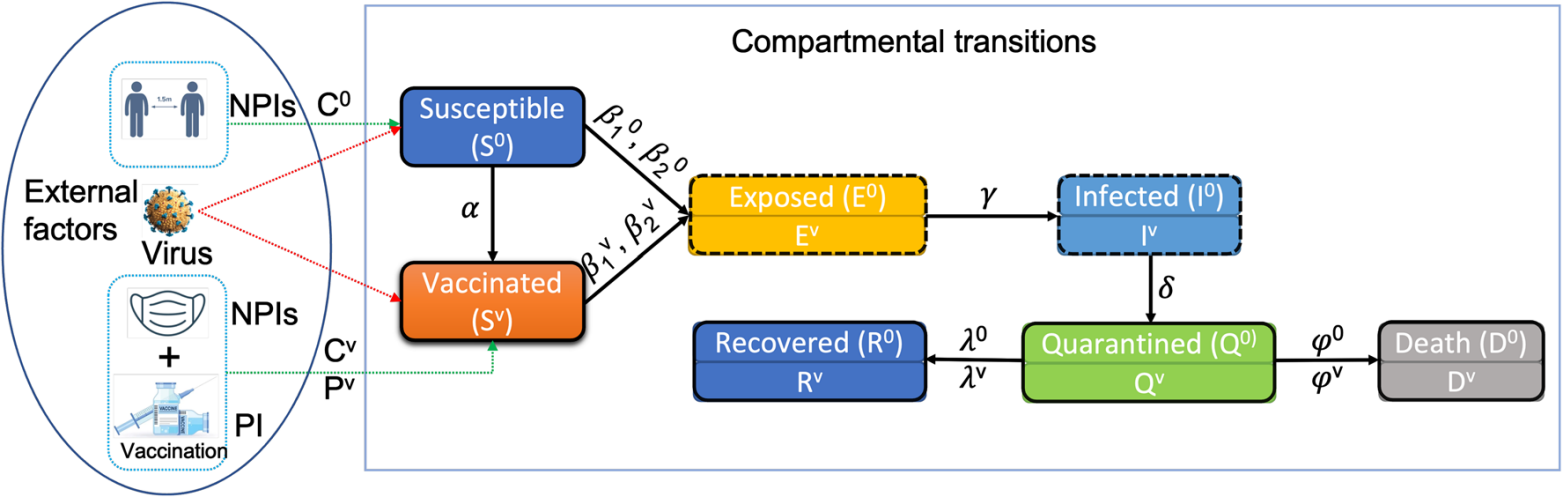
The structure and workflow of the model SEIQRD$^2$. In the external factor part, C$^0$ and C$^v$ represent NPIs events for the unvaccinated and vaccinated, respectively. P$^v$ stands for mass vaccination campaign. In the compartmental transition part, the status transitions among unvaccinated populations are described by the chain consisting of S$^0$, E$^0$, I$^0$, Q$^0$, R$^0$, and D$^0$. Similarly, the transition chain of the vaccinated consists of S$^v$, E$^v$, I$^v$, Q$^v$, R$^v$, and D$^v$. $\alpha$ is the daily vaccination rate, $\beta _{1}^{0}$ represents the effective transmission rate among Susceptible (S$^0$) caused by Infected (I$^0$), $\beta _{2}^{0}$ depicts the effective transmission rate among Susceptible (S$^0$) caused by Infected (I$^v$), $\beta _{1}^{v}$ is the effective transmission rate among Vaccinated(S$^v$) caused by Infected (I$^0$), $\beta _{2}^{v}$ is the effective transmission rate among Vaccinated (S$^v$) caused by Infected (I$^v$), 1/$\gamma$ is the incubation period, $\delta$ is the diagnosis rate, $\lambda$ is the recovery rate, and $\varphi$ is the death rate.

The model structure as shown in Fig. 7 illustrates the organization and arrangement of various model components in SEIQRD$^2$. The left part of Fig. 7 displays major external factors for COVID-19: virus, mass vaccination, and NPIs. The right panel outlines the dynamics of COVID-19. The compartmental transition part explicitly incorporates the impact of these external factors. For example, the SARS-CoV-2 virus was the cause of the COVID-19 pandemic, and its spread can be modelled and explained by an extended SEIR model as follows. In a mass vaccination campaign, the total population is typically categorized into two groups: those vaccinated and those who have not received the vaccine (i.e., unvaccinated). In addition, when a green pass policy is in effect, stricter NPIs are used to restrict the activities of the unvaccinated group than the vaccinated, which induces heterogeneous behaviors between these two groups. All these factors are captured by the two dependent SEIR-based chains in SEIQRD$^2$.

### Compartmental transitions

The compartmental transition part comprises two similar transition chains. The first chain is for the unvaccinated people. It consists of seven compartments: Susceptible (S$^0$), Exposed (E$^0$), Infected (I$^0$), Quarantined (Q$^0$), Recovered (R$^0$), and Death (D$^0$), which reflect the epidemiological compartmental transitions among the unvaccinated individuals during COVID-19. These components are presented in Fig. 7. Individuals in the Susceptible compartment (S$^0$) may get infected by those Infected (I$^0$) or Infected (I$^v$) at the effective transmission rate of $\beta _{1}^{0}$ and $\beta _{2}^{0}$, and become exposed, i.e., transited to the Exposed status (E$^0$). After a particular incubation period 1/$\gamma$, those exposed individuals (E$^0$) are transferred to the Infected compartment (I$^0$) and become infectious. Those infectious may be identified, confirmed and then transferred to the Quarantined compartment (Q$^0$) at a probability $\delta$. In Q$^0$, individuals either recover (R$^0$) at rate $\lambda$ or decease (D$^0$) at rate $\varphi$.

The other transition chain is for those vaccinated with green passes. Similarly, it consists of compartments Vaccinated (S$^v$), Exposed (E$^v$), Infected (I$^v$), Quarantined (Q$^v$), Recovered (R$^v$), and Death (D$^v$). The set of associated compartments depicts the dynamics of COVID-19 among green pass holders. Its transition process is also similar to those of unvaccinated people (i.e., the status S$^0$, E$^0$, I$^0$, Q$^0$, R$^0$, and D$^0$).

This bi-chain structure describes the status transitions among unvaccinated and vaccinated people during the COVID-19 pandemic and simultaneously considers their behavioral heterogeneity. Different from the existing epidemiological methods^12,13^, SEIQRD$^2$ takes a behavior informatics perspective to characterize the behaviors of vaccinated and unvaccinated groups and capture their interactions within and between groups. This explains how vaccination certification influences people’s behaviors differently and affects the resurgence of COVID-19 under the green pass policy. For instance, a susceptible person (S$^0$) may become infected by those vaccinated (I$^v$) at the probability of $\beta _{2}^{0}$. Besides, $\beta _{1}^{0}$ reflects the transmission rate at which the unvaccinated (I$^0$) spreads the virus to susceptible (S$^0$). The two dependent SEIR-based chains of SEIQRD$^2$ can be characterized by the following set of ordinary differential equations (ODE) (Eqs. 1–12). *N* refers to the population size.

1 $$\begin{aligned} \frac{dS^{0}}{dt}= & {} - \beta _{1}^{0} \frac{S^{0} I^{0}}{N}- \beta _{2}^{0} \frac{S^{0} I^{v}}{N} - \alpha N \end{aligned}$$

2 $$\begin{aligned} \frac{dS^{v}}{dt}= & {} - \beta _{1}^{v} \frac{S^{v} I^{0}}{N}- \beta _{2}^{v} \frac{S^{v} I^{v}}{N} + \alpha N \end{aligned}$$

3 $$\begin{aligned} \frac{dE^{0}}{dt}= & {} - \gamma E^{0} + \beta _{1}^{0} \frac{S^{0} I^{0}}{N} + \beta _{2}^{0} \frac{S^{0} I^{v}}{N} \end{aligned}$$

4 $$\begin{aligned} \frac{dE^{v}}{dt}= & {} - \gamma E^{v} + \beta _{1}^{v} \frac{S^{v} I^{0}}{N} + \beta _{2}^{v} \frac{S^{v} I^{v}}{N} \end{aligned}$$

5 $$\begin{aligned} \frac{dI^{0}}{dt}= & {} - \delta I^{0} + \gamma E^{0} \end{aligned}$$

6 $$\begin{aligned} \frac{dI^{v}}{dt}= & {} - \delta I^{v} + \gamma E^{v} \end{aligned}$$

7 $$\begin{aligned} \frac{dQ^{0}}{dt}= & {} - \lambda Q^{0} - \varphi Q^{0} + \delta I^{0} \end{aligned}$$

8 $$\begin{aligned} \frac{dQ^{v}}{dt}= & {} - \lambda Q^{v} - \varphi Q^{v} + \delta I^{v} \end{aligned}$$

9 $$\begin{aligned} \frac{dR^{0}}{dt}= & {} \lambda Q^{0} \end{aligned}$$

10 $$\begin{aligned} \frac{dR^{v}}{dt}= & {} \lambda Q^{v} \end{aligned}$$

11 $$\begin{aligned} \frac{dD^{0}}{dt}= & {} \varphi Q^{0} \end{aligned}$$

12 $$\begin{aligned} \frac{dD^{v}}{dt}= & {} \varphi Q^{v} \end{aligned}$$

$\alpha$, $\lambda$ and $\varphi$ are country specific parameters from real-world data described in Table 6, while $\beta _{1}^{0}$, $\beta _{2}^{0}$, $\beta _{1}^{v}$, $\beta _{2}^{v}$, $\gamma$ and $\delta$ are generated from the model.

**Table 6:** Model parameters for SEIQRD$^2$ and their associated values.

Parameters	Interpretation	Value
N	Population size	A fixed value—Country specific
$\alpha$	Daily vaccination rate	A series of values—Country specific
$\beta _{1}^{0}$	Effective transmission rate within unvaccinated	A series of values—Estimated
$\beta _{2}^{0}$	Effective transmission rate of vaccinated infected within unvaccinated	A series of values—Estimated
$\beta _{1}^{v}$	Effective transmission rate of unvaccinated infected within vaccinated	A series of values—Estimated
$\beta _2^{v}$	Effective transmission rate within vaccinated	A series of values—Estimated
$\gamma$	Incubation rate	A fixed value—Estimated
$\delta$	Quarantine rate	A fixed value—Estimated
$\lambda$	Recovery rate	A series of values—Country specific
$\varphi$	Mortality rate	A series of values—Country specific

### External factors

External factors, such as NPIs and mass vaccination, can significantly impact transitions between compartments. Therefore, it is necessary for SEIQRD$^2$ to quantify the effect of these NPI events and vaccination. Generally, the impact of such external factors is assessed by their role in changing the viral transmission speed. In SEIQRD$^2$, we measure the impact of external factors such as NPIs and vaccination on transmission rates. The effective transmission rates $\beta _{1}^{0}, \beta _{2}^{0}, \beta _{1}^{v}$ and $\beta _{2}^{v}$ are described by Eq. (13). Each transmission rate is a function of NPIs and vaccination.

13 $$\begin{aligned} \{\beta _{1}^{0}, \beta _{2}^{0}, \beta _{1}^{v}, \beta _{2}^{v} \} = \{ f_{1}^{0}, f_{2}^{0}, f_{1}^{v}, f_{2}^{v} \}(npis, vaccination) \end{aligned}$$

In theory, NPIs and vaccination are independent of each other. Therefore, Eq. (13) is transformed into Eq. (14).

14 $$\begin{aligned} \{\beta _{1}^{0}, \beta _{2}^{0}, \beta _{1}^{v}, \beta _{2}^{v} \} = \{ g_{1}^{0}(npis), g_{2}^{0}(npis), g_{1}^{v}(npis), g_{2}^{v}(npis)\} * h(vaccination) \end{aligned}$$

We further explain the functions of $g_{1}^{0}(npis)$, $g_{2}^{0}(npis)$, $g_{1}^{v}(npis)$, $g_{2}^{v}(npis)$, and *h*(*vaccination*).

During the COVID-19 pandemic, various NPIs were carried out to stop or mitigate the viral spread. Commonly, step functions are used to measure the effect of existing NPIs during a COVID-19 resurgence. In other words, at a given time segment, the effectiveness of NPIs is fixed as a constant value. The discrete values come from fitting the model, where *n* is the total number of NPIs-related events. $T_{i}$ represents the start time of the i-th NPI event, $T_{total}$ is the total number of days during a specific COVID-19 wave. For example, $R_{1,1}^{0}$ represents the impact of NPIs among the unvaccinated after the 1-th NPI event and before the 2-nd event, called the basic transmission rate. Therefore, the function of NPIs can be characterized as a set of discrete values by Eqs. (15)–(18).

15 $$\begin{aligned} g_{1}^{0}(npis) = \beta _{base,1}^{0}(t) = {\left\{ \begin{array}{ll} R_{1,0}^{0}, &{} T_{0}< t \leqslant T_{1} \\ R_{1,1}^{0}, &{} T_{1}< t \leqslant T_{2} \\ R_{1,i}^{0}, &{} T_{i}< t \leqslant T_{i+1} \\ R_{1,n}^{0}, &{} T_{n} < t \leqslant T_{0} + T_{total} \end{array}\right. } \end{aligned}$$

16 $$\begin{aligned} g_{2}^{0}(npis) = \beta _{base,2}^{0}(t) = \{ R_{2,1}^{0}, R_{2,2}^{0}, R_{2,3}^{0}, \ldots , R_{2,n}^{0} \} \end{aligned}$$

17 $$\begin{aligned} g_{1}^{v}(npis) = \beta _{base,1}^{v}(t) = \{ R_{1,1}^{v}, R_{1,2}^{v}, R_{1,3}^{v}, \ldots , R_{1,n}^{v} \} \end{aligned}$$

18 $$\begin{aligned} g_{2}^{v}(npis) = \beta _{base,2}^{v}(t) = \{ R_{2,1}^{v}, R_{2,2}^{v}, R_{2,3}^{v}, \ldots , R_{2,n}^{v} \} \end{aligned}$$

The effectiveness of vaccination is measured by Eq. (19). Many scientific studies explore the effectiveness of vaccines against the infection of COVID-19 over time. In this work, we use the results about the Pfizer-BioNTech (BNT162b2) COVID-19 vaccine in Ref.^44^ to compute the effectiveness of vaccination during COVID-19 resurgence because the Pfizer vaccination took the highest proportion around the world, including the chosen three countries: Greece ($>70\%$), Austria ($>60\%$), and Israel ($>80\%$). *M* refers to the total population of the vaccinated until the t-th day. $t_{0}$ is the start time of mass vaccination. *Num*(*j*) is the number of the vaccinated people on the *j*-th day. $Eff(t-j)$ refers to the vaccine effect on the infection $(t-j)$ days after vaccination. In Eq. (20), the coefficients $\{0.775, 0.732, 0.696, 0.517, 0.225, 0.173 \}$ correspond to the results in Ref.^44^. To simplify the modeling, we assume the booster’s effectiveness follows the same diminishing efficacy trend in Ref.^44^. Besides, the assumption will not significantly change the general results of SEIQRD$^2$ about NPIs. Because the impact of NPIs is described by step functions in Eqs. (15)–(18), which are independent of the effectiveness of vaccination represented by the ramp function in Eq. (19).

19 $$\begin{aligned} h(vaccination) = h(t) = 1 - \frac{1}{M} \sum _{j=t_{0}}^{t} Num(j) Eff(t-j) \end{aligned}$$

20 $$\begin{aligned} Eff(t-j) = {\left\{ \begin{array}{ll} 0.775, &{} 0< t-j \leqslant 30 \\ 0.732, &{} 30< t-j \leqslant 60 \\ 0.696, &{} 60< t-j \leqslant 90\\ 0.517, &{} 90< t-j \leqslant 120\\ 0.225, &{} 120< t-j \leqslant 150\\ 0.173, &{} 150 < t-j \end{array}\right. } \end{aligned}$$

Then, Eq. (14) can be converted to Eqs. (21–24).

21 $$\begin{aligned} \beta _{1}^{0}(t)= & {} \beta _{base,1}^{0}(t) \end{aligned}$$

22 $$\begin{aligned} \beta _{2}^{0}(t)= & {} \beta _{base,2}^{0}(t) \end{aligned}$$

23 $$\begin{aligned} \beta _{1}^{v}(t)= & {} \beta _{base,1}^{v}(t) * h(t)= {\left\{ \begin{array}{ll} R_{1,0}^{v} * [1 - \frac{1}{M} \sum _{j=t_{0}}^{t} Num(j) Eff(t-j)], &{} T_{0}< t \leqslant T_{1} \\ R_{1,1}^{v} * [1 - \frac{1}{M} \sum _{j=t_{0}}^{t} Num(j) Eff(t-j)], &{} T_{1}< t \leqslant T_{2} \\ R_{1,i}^{v} * [1 - \frac{1}{M} \sum _{j=t_{0}}^{t} Num(j) Eff(t-j)], &{} T_{i}< t \leqslant T_{i+1}\\ R_{1,n}^{v} * [1 - \frac{1}{M} \sum _{j=t_{0}}^{t} Num(j) Eff(t-j)], &{} T_{n} < t \leqslant T_{0} + T_{total} \end{array}\right. } \end{aligned}$$

24 $$\begin{aligned} \beta _{2}^{v}(t)= & {} \beta _{base,2}^{v}(t) * h(t) \end{aligned}$$

### Estimation

We solve the model with a nonlinear data-fitting approach that minimizes a least squares error function as shown in Eq. (25), where *F* represents our model, ${\textbf{x}}$ denotes the input data (*Q*, *R* and *D*), provided by the integration of the ordinary differential equation (ODE) system (Eqs. 1–12) and solved by a fourth-order Runge-Kutta method. *y* denotes an observation (i.e., the reported active case number). $\mathbf {\theta }$ refers to all parameters ($\alpha$, $\beta _{1}^{0}$, $\beta _{2}^{0}$, $\beta _{1}^{v}$, $\beta _{2}^{v}$, $\gamma$, $\delta$, $\lambda$, $\varphi$) that are inferred by Eqs. (1)–(12). $T_{total}$ is the total number of days.

Model parameters are based on formal estimation. In order to obtain the simulation results, we take the classical optimization algorithm (the least-squares method) to fit actual quarantined cases with a given objective function in Eq. (25). In Eq. (25), $y_{j}$ is the actual quarantined case in the j-th day.

25 $$\begin{aligned} \min _{\theta }\Vert F(\mathbf {\theta }, {\textbf{x}}) - y\Vert ^2 = \min _{\theta } \sum _j^{T_{total}} (F(\mathbf {\theta }, x) - y_{j})^2 \end{aligned}$$

This function requires initial values for optimization. We randomly set the initial values for the unknown parameter set $\mathbf {\theta }$ with the lower bound 0. The representative measure of the optimal set of parameters is obtained with up to 10,000 optimization iterations under the initial values.

### Evaluation

The MAPE defined in Eq. (26) is also a key metric to assess the performance of SEIQRD$^2$ in predicting the dynamics of COVID-19. $y_{j}$ is the actual quarantined case or total deaths on the j$^{th}$ day. $\bar{y_{j}}$ is the prediction of SEIQRD$^2$ about quarantined cases or total deaths. $T_{total}$ is the total number of days. For instance, the prediction of the next 14-day quarantined cases and total deaths is measured by the MAPE of the simulation results.

26 $$\begin{aligned} MAPE = \frac{1}{T_{total}} \sum _{j=1}^{T_{total}}\frac{|y_{j} - \bar{y_{j}}|}{y_{j}} \end{aligned}$$

### Explanatory model for transmission rates

In the literature, the SEIR model and its modified versions are frequently utilized in epidemiology^5^. One of the most obvious reasons is its strong explainability from the epidemic perspective. They capture important epidemiological information, such as transmission rate and incubation period. These parameters could give some insights into the dynamics of infectious diseases. The four transmission rates $\beta _{1}^{0}$, $\beta _{2}^{0}$, $\beta _{1}^{v}$, and $\beta _{2}^{v}$ in the SEIQRD$^2$ model provide a more accurate representation of the COVID-19 pandemic compared to other compartmental models. These rates consider the vaccination status of the population. Compared to the single transmission rate of SEIR and its modified versions, the four transmission rates in SEIQRD$^2$ are effective in depicting the distinct spread of COVID-19 across different groups vaccinated or unvaccinated and their interactions within and between groups. This behavioral approach also provides decision-makers with a deeper insight into how the COVID-19 transmits in vaccinated versus unvaccinated groups. For instance, when both $\beta _{1}^{0}$ and $\beta _{2}^{v}$ rise, it indicates a decrease of NPIs or the emergence of a more contagious virus strain. Besides, combined with the decrease of $\beta _{1}^{0}$, the increase of $\beta _{2}^{v}$ points out that the phenomenon may be caused by over-relaxation interventions for green pass holders or declined vaccine effectiveness. Furthermore, $\beta _{2}^{0}$ and $\beta _{1}^{v}$ capture the interactions between unvaccinated and vaccinated groups. For instance, the rise of $\beta _{2}^{0}$ shows that the activities of those vaccinated increase the risk of unvaccinated people being infected. Accordingly, it would be reasonable to enforce appropriate restrictions on those vaccinated. With these four rates, SEIQRD$^2$ can provide a more comprehensive understanding of the dynamics of COVID-19 within and between different groups. This understanding offers reasonable adjustments to existing control measures.

The effective transmission rates measure the joint impact of NPIs and mass vaccination on the COVID-19 transmission. In contrast, its corresponding basic transmission rates $\{ \beta _{base,1}^{0}, \beta _{base,2}^{0}, \beta _{base,1}^{v}, \beta _{base,2}^{v} \}$ consider the impact of NPIs alone. It is essential to assess the fundamental transmission rates to judge the efficacy of existing NPIs when mass vaccination becomes accessible. It can be utilized to simultaneously monitor the impact of NPIs and vaccination, providing quantitative measures to adjust NPI restrictions and mass vaccination strategies.

## Data Availability

The datasets generated and/or analysed during the current study are available from the Johns Hopkins University research database (github.com/CSSEGISandData/COVID-19), and Our World in Data (ourworldindata. com).

## Code availibility

*Accession codes and data* (https://github.com/lzxiaohu/SEIQRD2).
